# 3-(2-Hy­droxy­eth­yl)-1-(4-nitro­phen­yl)-1*H*-imidazol-3-ium bromide

**DOI:** 10.1107/S2414314624011386

**Published:** 2024-11-28

**Authors:** Halliru Ibrahim, Sizwe J. Zamisa, Muhammad D. Bala, Pinkie Ntola, Holger B. Friedrich

**Affiliations:** a Department of Chemistry, Durban University of Technology, PO Box 1334, Durban, 4000, South Africa; b School of Chemistry and Physics, University of KwaZulu-Natal, Private Bag X54001, Durban, 4000, South Africa

**Keywords:** crystal structure, imidazolium salt, hydrogen-bonding

## Abstract

The mol­ecular structure of the title salt, C_11_H_12_N_3_O_3_^+^·Br^−^, reveals near co-planarity between the the imidazole and 4-nitro­benzene moieties with a dihedral angle of 8.99 (14)° between their planes. A prominent feature in the mol­ecular packing is the bromide anion acting as a double acceptor for O—H⋯Br and C—H⋯Br hydrogen-bonds, leading to a linear chain propagating along [110]. The crystal studied was refined as an inversion twin, with the minor component = 0.081 (8).

## Abstract 2

The crystal structure of an imidazolium bromide salt is described.

## Graphical abstract

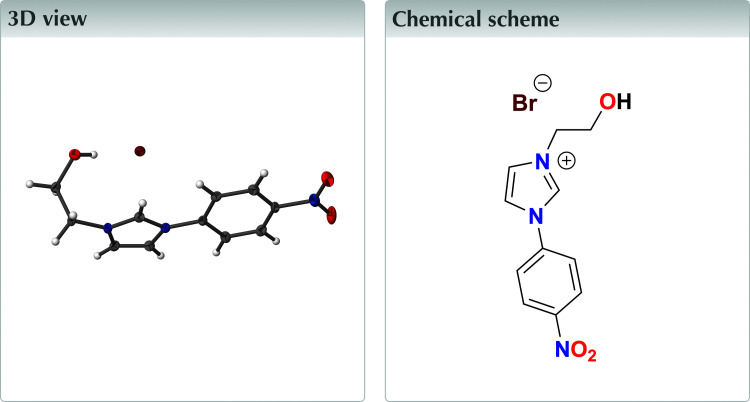

## Structure description

The title crystal is an imidazolium bromide salt based on a 1-(4-nitro­phen­yl)-1*H*-imidazol-3-yl moiety (Ibrahim & Bala, 2016 ▸; Illam *et al.*, 2021 ▸), which is functionalized at the 1,3-diazole wingtip with a 2-hy­droxy­ethyl group. Unlike analogues with a fused 1*H*-benzo[*d*] backbone (Kumar *et al.*, 2015 ▸), derivatives of the title salt with the 1*H*-imidazol-3-yl moiety do not show any potential as chemodosimeters. The incorporation of oxygen-containing functionalities in the design of these imidazolium salts is motivated by the desire to increase the solubility of the ligand/precursor in common solvents (Garrison & Young, 2005 ▸), and, upon coordination, to enhance the electron density around the metal, thereby stabilizing the metal center during a catalytic cycle (Simpson *et al.*, 2015 ▸). We recently explored the potential of such NO_2_-functionalized imidazolyl­idene–Co^II^/Ni^II^ complexes as viable green catalysts for aryl C—N coupling reactions of aryl amines with aryl bromides (Ibrahim & Bala, 2016 ▸). In a continuation of this work designed to develop new derivatives with superior catalytic abilities, the title compound was synthesized and analyzed by X-ray crystallography.

**Figure 1:**
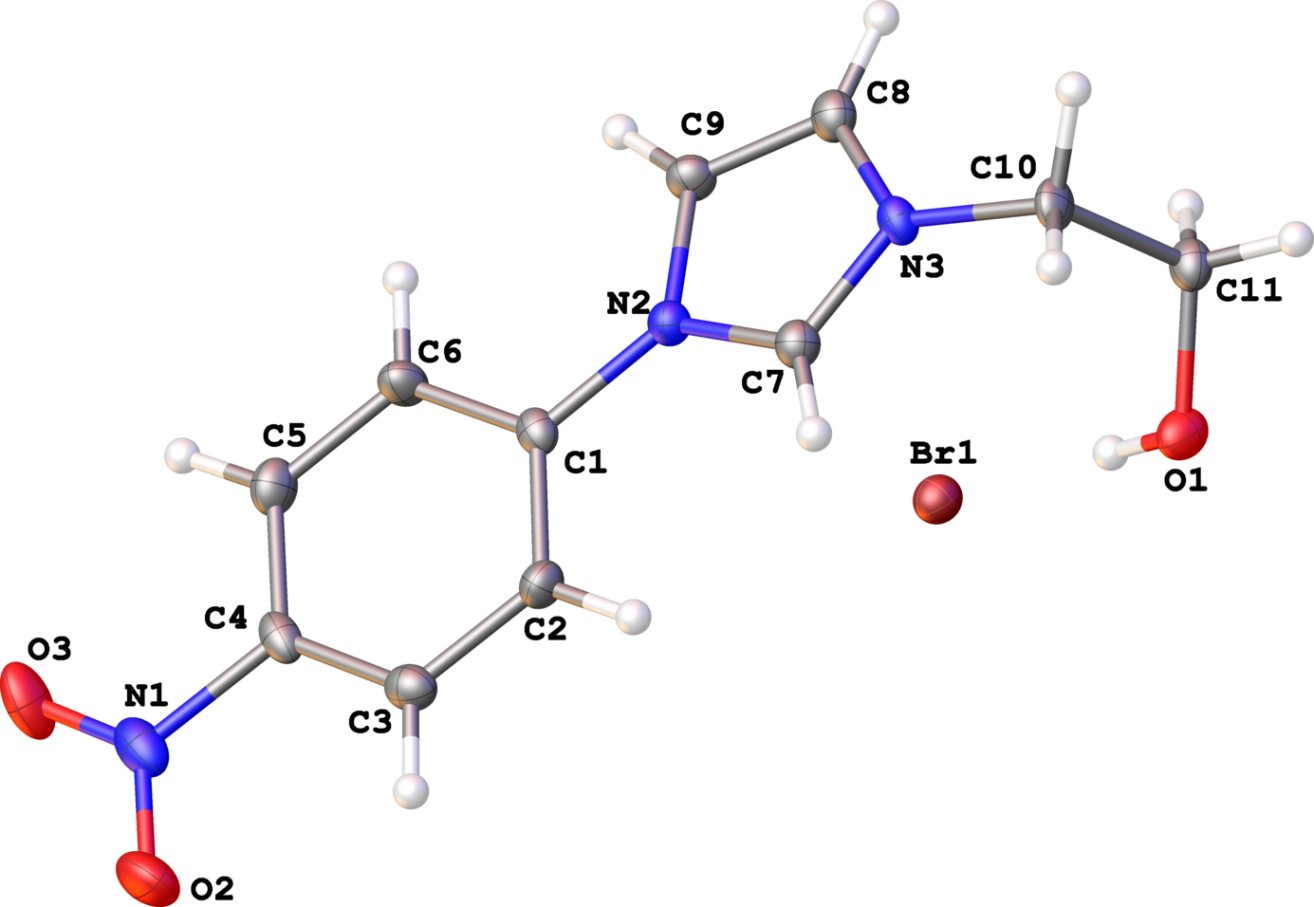
The asymmetric unit of the title compound showing the atom-labeling scheme and displacement ellipsoids at the 50% probability level.

**Figure 2:**
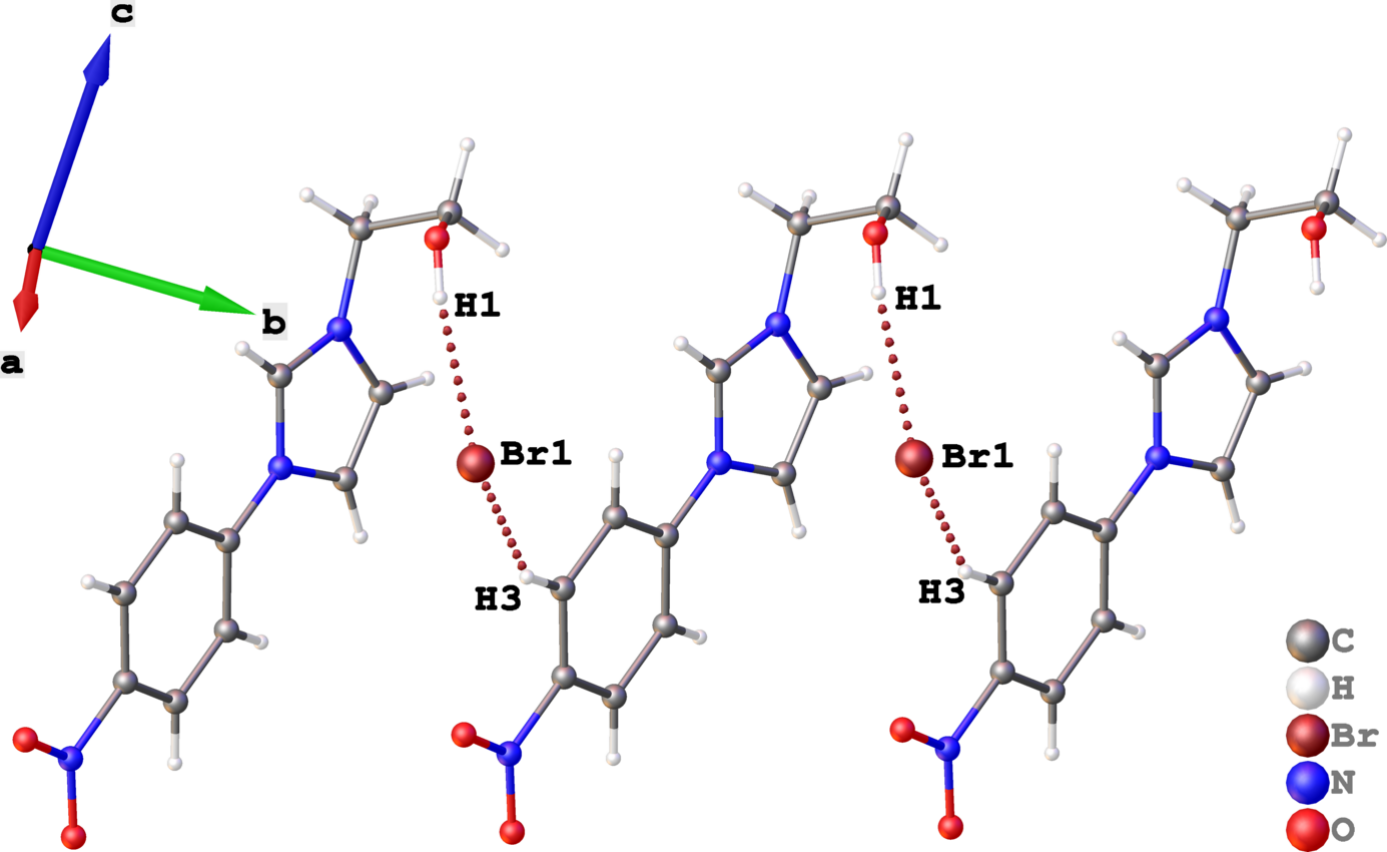
Representation of inter­molecular O1—H1⋯Br1 and C3—H3⋯Br1 hydrogen bonds (brown dotted bonds) within the crystal of the title compound.

The asymmetric unit of the title salt comprises an imidazolyl cation and a bromide anion (Fig. 1 ▸). The conformation of the cationic species is such that the dihedral angle between the imidazole and 4-nitro­benzene planes is 8.99 (14)° while the orientation of the ethanolyl side is almost orthogonal with respect to the imidazole plane [C7—N3—C10—C11 torsion angle = 95.1 (4)°]. A prominent feature of the mol­ecular packing relates to the bromide anion acting as a double acceptorm with the hydroxyl-H1 atom and the H3 atom of a neighboring 4-nitro­phenyl moiety to form a linear supra­molecular chain propagatingalong [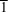
10] (Table 1 ▸; Fig. 2 ▸).

## Synthesis and crystallization

The title compound was synthesized with a slight modification of the reported protocol (Ibrahim & Bala, 2016 ▸). A mixture of *N*-*p*-nitro­phenyl imidazole (0.5 g, 0.003 mol) and 2-bromo­ethanol (0.56 g, 0.005 mol; 0.35 ml, ρ = 1.76 g cm^−3^, 95%) was refluxed overnight in aceto­nitrile under an inert di­nitro­gen atmosphere. Removal of the solvent followed by washing with ethyl acetate afforded a yellow precipitate, which yielded the title salt as an air-stable yellow solid after drying *in vacuo*. Slow diffusion of diethyl ether into a methano­lic solution of the isolated title salt afforded suitable single crystals for the X-ray diffraction analysis. Yield: 0.70 g, 0.002 mol, 85.7%. m.p. 475–477 K. ^1^H NMR (400 MHz, DMSO-*d*_6_): δ 10.03 (*s*, 1H, NCHN), 8.52 (*d*, *J* = 9.0 Hz, 2H, CH_(phen­yl)_), 8.47 (*d*, *J* = 1.6 Hz, 1H, CH_(imidazol­yl)_), 8.11 (*d*, *J* = 9.1 Hz, 2H, CH_(benz­yl)_), 8.06 (*s*, 1H, CH_(imidazol­yl)_), 4.33 (*t*, *J* = 10.0 Hz, 2H, CH_2(ethano­yl)_), 3.84 (*t*, *J* = 10.0 Hz, 2H, CH_2_(hy­droxy­ethy), 3.35 (*s*, *b*, 1H, OH(hy­droxy­eth­yl)). ^13^C NMR (100 MHz, DMSO-*d*_6_): δ 147.5 (NCN), 139.2, 136.4, 125.6, 124.1, 122.9, 120.9, 59.1 (CH_2_), 52.4 (CH_2_). FTIR (ν(O—H) 3243, ν(aryl C—H) 3090, ν(alkyl C—H) 2958, ν(C=N) 1552, ν(NO_2_) 1522 & 1341, ν(C—O) 1222, ν(phen­yl) 854 cm^−1^. LRMS-ES^+^: *m*/*z* (%) 234.0550 (100) [(*M* − Br)]^+^.

## Refinement

Table 2 ▸ provides a summary of the crystal data, data collection and structure refinement details. The structure was refined as an inversion twin, with the minor component = 0.081 (8).

## Supplementary Material

Crystal structure: contains datablock(s) I. DOI: 10.1107/S2414314624011386/tk4113sup1.cif

Structure factors: contains datablock(s) I. DOI: 10.1107/S2414314624011386/tk4113Isup2.hkl

Supporting information file. DOI: 10.1107/S2414314624011386/tk4113Isup3.cml

CCDC reference: 2404555

Additional supporting information:  crystallographic information; 3D view; checkCIF report

## Figures and Tables

**Table 1:** Hydrogen-bond geometry (Å, °)

*D*—H⋯*A*	*D*—H	H⋯*A*	*D*⋯*A*	*D*—H⋯*A*
O1—H1⋯Br1	0.84	2.42	3.2509 (19)	171
C3—H3⋯Br1^i^	0.95	2.71	3.572 (2)	151

Symmetry code: (i) 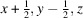.

**Table 2:** Experimental details

Crystal data	
Chemical formula	C_11_H_12_N_3_O_3_^+^·Br^−^
*M*_r_	314.15
Crystal system, space group	Monoclinic, *Cc*
Temperature (K)	100
*a*, *b*, *c* (Å)	6.4352 (4), 12.2697 (11), 15.5936 (10)
β (°)	90.290 (3)
*V* (Å^3^)	1231.22 (16)
*Z*	4
Radiation type	Mo *K*α
μ (mm^−1^)	3.34
Crystal size (mm)	0.38 × 0.21 × 0.14

Data collection	
Diffractometer	Bruker SMART APEXII area detector
Absorption correction	Multi-scan (*SADABS*; Krause *et al.*, 2015 ▸)
*T*_min_, *T*_max_	0.661, 0.746
No. of measured, independent and observed [*I* > 2σ(*I*)] reflections	9019, 3051, 2930
*R*_int_	0.019
(sin θ/λ)_max_ (Å^−1^)	0.673

Refinement	
*R*[*F*^2^ > 2σ(*F*^2^)], *wR*(*F*^2^), *S*	0.017, 0.037, 1.03
No. of reflections	3051
No. of parameters	165
No. of restraints	2
H-atom treatment	H-atom parameters constrained
Δρ_max_, Δρ_min_ (e Å^−3^)	0.36, −0.23
Absolute structure	Refined as an inversion twin
Absolute structure parameter	0.081 (8)

Computer programs: *COSMO* and *SAINT* (Bruker, 2010 ▸), *SHELXT2013* (Sheldrick, 2015*a* ▸), *SHELXL2018/3* (Sheldrick, 2015*b* ▸) and *OLEX2* (Dolomanov *et al.*, 2009 ▸).

## full crystallographic data

### (I) Crystal data

**Table:**

C_11_H_12_N_3_O^3+^·Br^−^	*F*(000) = 632
*M**_r_* = 314.15	*D*_x_ = 1.695 Mg m^−^^3^
Monoclinic, *C**c*	Mo *K*α radiation, λ = 0.71073 Å
*a* = 6.4352 (4) Å	Cell parameters from 6616 reflections
*b* = 12.2697 (11) Å	θ = 3.3–28.5°
*c* = 15.5936 (10) Å	µ = 3.34 mm^−^^1^
β = 90.290 (3)°	*T* = 100 K
*V* = 1231.22 (16) Å^3^	Slab, light yellow
*Z* = 4	0.38 × 0.21 × 0.14 mm

### (I) Data collection

**Table:**

Bruker SMART APEXII area detector diffractometer	2930 reflections with *I* > 2σ(*I*)
Detector resolution: 7.9 pixels mm^-1^	*R*_int_ = 0.019
ω and φ scans	θ_max_ = 28.6°, θ_min_ = 2.6°
Absorption correction: multi-scan (SADABS; Krause *et al.*, 2015)	*h* = −8→8
*T*_min_ = 0.661, *T*_max_ = 0.746	*k* = −16→16
9019 measured reflections	*l* = −20→20
3051 independent reflections	

### (I) Refinement

**Table:**

Refinement on *F*^2^	Secondary atom site location: difference Fourier map
Least-squares matrix: full	Hydrogen site location: inferred from neighbouring sites
*R*[*F*^2^ > 2σ(*F*^2^)] = 0.017	H-atom parameters constrained
*wR*(*F*^2^) = 0.037	*w* = 1/[σ^2^(*F*_o_^2^) + (0.0076*P*)^2^] where *P* = (*F*_o_^2^ + 2*F*_c_^2^)/3
*S* = 1.03	(Δ/σ)_max_ < 0.001
3051 reflections	Δρ_max_ = 0.36 e Å^−^^3^
165 parameters	Δρ_min_ = −0.22 e Å^−^^3^
2 restraints	Absolute structure: Refined as an inversion twin
Primary atom site location: structure-invariant direct methods	Absolute structure parameter: 0.081 (8)

### (I) Special details

**Table:**

Geometry. All esds (except the esd in the dihedral angle between two l.s. planes) are estimated using the full covariance matrix. The cell esds are taken into account individually in the estimation of esds in distances, angles and torsion angles; correlations between esds in cell parameters are only used when they are defined by crystal symmetry. An approximate (isotropic) treatment of cell esds is used for estimating esds involving l.s. planes.
Refinement. Refined as a 2-component inversion twin.

### (I) Fractional atomic coordinates and isotropic or equivalent isotropic displacement parameters (Å^2^)

**Table:**

	*x*	*y*	*z*	*U*_iso_*/*U*_eq_	
Br1	0.52235 (5)	0.56771 (2)	0.24805 (3)	0.01782 (6)	
C2	0.6133 (4)	0.2573 (2)	0.12029 (17)	0.0170 (5)	
H2	0.588284	0.236441	0.177996	0.020*	
O2	1.1242 (3)	0.15187 (18)	−0.01212 (14)	0.0336 (6)	
O1	0.2180 (3)	0.45365 (14)	0.38751 (12)	0.0204 (4)	
H1	0.298129	0.475858	0.348926	0.031*	
C11	0.0084 (5)	0.4666 (3)	0.3601 (2)	0.0178 (7)	
H11A	−0.007513	0.538803	0.332540	0.021*	
H11B	−0.083443	0.464705	0.410782	0.021*	
C10	−0.0603 (5)	0.3781 (2)	0.29685 (18)	0.0165 (6)	
H10A	−0.035231	0.305423	0.322381	0.020*	
H10B	−0.211178	0.385282	0.285381	0.020*	
N3	0.0538 (4)	0.38742 (19)	0.21627 (15)	0.0140 (5)	
C7	0.2242 (4)	0.33242 (19)	0.19668 (16)	0.0160 (5)	
H7	0.287958	0.278530	0.231755	0.019*	
N2	0.2926 (3)	0.36462 (15)	0.11964 (13)	0.0141 (4)	
C1	0.4742 (4)	0.32634 (19)	0.07739 (15)	0.0145 (5)	
C3	0.7879 (4)	0.2196 (2)	0.07779 (17)	0.0185 (5)	
H3	0.883162	0.171970	0.105583	0.022*	
C4	0.8207 (3)	0.2526 (2)	−0.00536 (18)	0.0164 (5)	
N1	1.0073 (4)	0.21336 (18)	−0.05093 (16)	0.0229 (5)	
O3	1.0345 (4)	0.24477 (17)	−0.12456 (14)	0.0306 (5)	
C8	0.0106 (6)	0.4587 (3)	0.1501 (2)	0.0178 (7)	
H8	−0.102340	0.508493	0.147718	0.021*	
C6	0.5101 (4)	0.3571 (2)	−0.00708 (17)	0.0182 (6)	
H6	0.414163	0.403271	−0.035965	0.022*	
C5	0.6850 (4)	0.3206 (2)	−0.04886 (17)	0.0193 (5)	
H5	0.711834	0.341669	−0.106364	0.023*	
C9	0.1575 (4)	0.44526 (18)	0.08943 (17)	0.0168 (5)	
H9	0.166987	0.483219	0.036499	0.020*	

### (I) Atomic displacement parameters (Å^2^)

**Table:**

	*U*^11^	*U*^22^	*U*^33^	*U*^12^	*U*^13^	*U*^23^
Br1	0.01789 (10)	0.01860 (10)	0.01696 (10)	−0.00135 (15)	−0.00015 (7)	0.00074 (15)
C2	0.0190 (14)	0.0193 (12)	0.0127 (12)	0.0002 (11)	−0.0003 (11)	0.0011 (9)
O2	0.0239 (12)	0.0384 (13)	0.0386 (14)	0.0131 (10)	0.0002 (10)	−0.0074 (10)
O1	0.0181 (9)	0.0268 (9)	0.0163 (9)	−0.0040 (7)	0.0012 (7)	0.0031 (7)
C11	0.0188 (16)	0.0178 (14)	0.0169 (15)	−0.0021 (12)	0.0021 (12)	−0.0041 (12)
C10	0.0163 (14)	0.0203 (14)	0.0130 (13)	−0.0032 (11)	0.0056 (11)	−0.0004 (11)
N3	0.0143 (12)	0.0110 (10)	0.0167 (11)	−0.0017 (9)	0.0034 (9)	−0.0022 (8)
C7	0.0169 (13)	0.0154 (11)	0.0157 (12)	−0.0011 (9)	0.0011 (9)	0.0000 (9)
N2	0.0153 (10)	0.0128 (9)	0.0144 (10)	−0.0004 (8)	0.0006 (8)	−0.0001 (7)
C1	0.0143 (12)	0.0127 (11)	0.0165 (13)	−0.0017 (9)	0.0018 (10)	−0.0033 (9)
C3	0.0176 (13)	0.0158 (12)	0.0219 (13)	0.0014 (10)	−0.0021 (10)	−0.0017 (10)
C4	0.0134 (15)	0.0148 (10)	0.0211 (12)	−0.0034 (12)	0.0045 (12)	−0.0060 (9)
N1	0.0181 (12)	0.0186 (11)	0.0320 (14)	−0.0028 (9)	0.0050 (10)	−0.0081 (10)
O3	0.0319 (13)	0.0271 (11)	0.0329 (12)	0.0010 (9)	0.0190 (10)	0.0008 (9)
C8	0.0196 (16)	0.0170 (14)	0.0170 (15)	−0.0008 (12)	0.0011 (12)	−0.0026 (11)
C6	0.0178 (14)	0.0189 (13)	0.0180 (14)	0.0042 (11)	0.0007 (11)	0.0037 (10)
C5	0.0226 (14)	0.0194 (12)	0.0159 (12)	−0.0037 (10)	0.0034 (10)	0.0009 (10)
C9	0.0182 (12)	0.0143 (11)	0.0179 (13)	0.0018 (9)	−0.0006 (10)	0.0022 (9)

### (I) Geometric parameters (Å, º)

**Table:**

C2—H2	0.9500	C7—N2	1.341 (3)
C2—C1	1.400 (3)	N2—C1	1.424 (3)
C2—C3	1.387 (4)	N2—C9	1.398 (3)
O2—N1	1.224 (3)	C1—C6	1.391 (3)
O1—H1	0.8400	C3—H3	0.9500
O1—C11	1.421 (4)	C3—C4	1.376 (4)
C11—H11A	0.9900	C4—N1	1.479 (3)
C11—H11B	0.9900	C4—C5	1.383 (4)
C11—C10	1.530 (4)	N1—O3	1.225 (3)
C10—H10A	0.9900	C8—H8	0.9500
C10—H10B	0.9900	C8—C9	1.351 (5)
C10—N3	1.463 (3)	C6—H6	0.9500
N3—C7	1.325 (3)	C6—C5	1.378 (4)
N3—C8	1.379 (4)	C5—H5	0.9500
C7—H7	0.9500	C9—H9	0.9500
			
C1—C2—H2	120.3	C2—C1—N2	120.2 (2)
C3—C2—H2	120.3	C6—C1—C2	120.5 (2)
C3—C2—C1	119.4 (2)	C6—C1—N2	119.3 (2)
C11—O1—H1	109.5	C2—C3—H3	120.6
O1—C11—H11A	109.1	C4—C3—C2	118.7 (2)
O1—C11—H11B	109.1	C4—C3—H3	120.6
O1—C11—C10	112.7 (3)	C3—C4—N1	119.1 (2)
H11A—C11—H11B	107.8	C3—C4—C5	122.7 (2)
C10—C11—H11A	109.1	C5—C4—N1	118.2 (2)
C10—C11—H11B	109.1	O2—N1—C4	117.5 (2)
C11—C10—H10A	109.5	O2—N1—O3	124.6 (3)
C11—C10—H10B	109.5	O3—N1—C4	117.9 (2)
H10A—C10—H10B	108.1	N3—C8—H8	126.0
N3—C10—C11	110.7 (2)	C9—C8—N3	107.9 (3)
N3—C10—H10A	109.5	C9—C8—H8	126.0
N3—C10—H10B	109.5	C1—C6—H6	120.0
C7—N3—C10	125.3 (2)	C5—C6—C1	120.0 (2)
C7—N3—C8	108.3 (3)	C5—C6—H6	120.0
C8—N3—C10	126.3 (3)	C4—C5—H5	120.7
N3—C7—H7	125.3	C6—C5—C4	118.6 (2)
N3—C7—N2	109.4 (2)	C6—C5—H5	120.7
N2—C7—H7	125.3	N2—C9—H9	126.7
C7—N2—C1	126.3 (2)	C8—C9—N2	106.7 (2)
C7—N2—C9	107.7 (2)	C8—C9—H9	126.7
C9—N2—C1	126.0 (2)		
			
C2—C1—C6—C5	−0.8 (4)	N2—C1—C6—C5	−179.9 (2)
C2—C3—C4—N1	179.6 (2)	C1—C2—C3—C4	0.8 (4)
C2—C3—C4—C5	−1.0 (4)	C1—N2—C9—C8	−178.1 (2)
O1—C11—C10—N3	−66.2 (3)	C1—C6—C5—C4	0.6 (4)
C11—C10—N3—C7	95.1 (4)	C3—C2—C1—N2	179.2 (2)
C11—C10—N3—C8	−80.8 (3)	C3—C2—C1—C6	0.0 (4)
C10—N3—C7—N2	−177.1 (2)	C3—C4—N1—O2	0.6 (3)
C10—N3—C8—C9	177.1 (2)	C3—C4—N1—O3	−179.2 (2)
N3—C7—N2—C1	178.5 (2)	C3—C4—C5—C6	0.2 (4)
N3—C7—N2—C9	0.4 (3)	N1—C4—C5—C6	179.7 (2)
N3—C8—C9—N2	−0.3 (3)	C8—N3—C7—N2	−0.6 (3)
C7—N3—C8—C9	0.6 (3)	C5—C4—N1—O2	−178.9 (2)
C7—N2—C1—C2	−7.5 (3)	C5—C4—N1—O3	1.4 (4)
C7—N2—C1—C6	171.7 (2)	C9—N2—C1—C2	170.3 (2)
C7—N2—C9—C8	−0.1 (3)	C9—N2—C1—C6	−10.6 (3)

### (I) Hydrogen-bond geometry (Å, º)

**Table:**

*D*—H···*A*	*D*—H	H···*A*	*D*···*A*	*D*—H···*A*
O1—H1···Br1	0.84	2.42	3.2509 (19)	171
C3—H3···Br1^i^	0.95	2.71	3.572 (2)	151

Symmetry code: (i) *x*+1/2, *y*−1/2, *z*.
